# Comparison of pregnancy outcome in half-dose Triptorelin and short-acting Decapeptyl in long protocol in ART cycles: A randomized clinical trial

**Published:** 2013-02

**Authors:** Maryam Eftekhar, Elham Rahmani, Farnaz Mohammadian

**Affiliations:** 1 *Research and Clinical Center for Infertility, Shahid Sadoughi University of Medical Sciences, Yazd, Iran.*; 2 *Department of Obstetrics and Gynecology, Bushehr University of Medical Sciences, Bushehr, Iran.*; 3 *Department of Obstetrics and Gynecology, Zanjan University of Medical Sciences, Zanjan, Iran.*

**Keywords:** *Infertility*, *GnRH agonist*, *In vitro fertilization*, *Pregnancy outcome*, *Decapeptyl*

## Abstract

**Background: **Gonadotrophin-releasing hormone (GnRH) agonist is used for controlling ovarian stimulation in assisted reproductive technology (ART) cycles which has some benefits.

**Objective:** To compare the efficacy of two different formulations of GnRH agonist: short-acting and long-acting, for ART protocols.

**Materials and Methods:** In a prospective randomized study, one hundred women who underwent ART cycles were randomly divided into two groups. In group I, the patients received one single injection of 1.87 mg Triptorelin in previous mid-luteal phase. In group II, Decapeptyl 0.1 mg per day started from previous mid-luteal phase. Pregnancy outcome in in vitro fertilization (IVF) cycle was compared between two groups.

**Results:** There were no statistically significant differences in the number of retrieved oocyte (p=0.545), fertilization (p=0.876), implantation (p=0.716) and pregnancy rate (p=0.727) between the two groups.

**Conclusion:** There were not any advantages in IVF outcome between half-dose long-acting and short-acting GnRH agonist groups in ART cycle.

## Introduction

Gonadotrophin-releasing hormone agonist (GnRh) agonist is used for many women in order to control ovarian stimulation (COH) in assisted reproductive technology )ART( cycles. The benefits of GnRH agonist in COH include: preventing premature LH surge and luteinization, decreasing the cancellation rate of cycles, improving follicular recruitment, increasing the number of follicles, good quality oocyte and embryos. GnRH agonist is routinely used in long protocols (1-3). Two types of GnRH agonist are available which are used for desensitization of hypothesis in ART cycles: one type, short-acting GnRH agonist and another long-acting (depot form) (2, 4, 5). 

The pituitary desensitization time is no difference between long and short-acting GnRH-a, but the duration of short GnRH agonist action is shorter and it allows a quicker recovery of pituitary gonadotropin secretion after its withdrawal. 

Depot form of GnRH agonist has a longer half-life; however, there is concern about its unfavorable effects on embryo during early gestation (6-8). When short GnRH agonist is used for long protocol, it needs repeated daily administration for several days so this protocol is less acceptable by patients. Patient’s tolerance is better by single administration of depot from of GnRH agonist (5, 7, 9).

The purpose of this study was to compare pregnancy outcome in two types of long GnRH agonist protocols: half-dose long-acting and short-acting GnRH agonist in ART cycles.

## Materials and methods

The study was conducted in a prospective randomized manner from January 2010 to December 2011 at Yazd Research and Clinical Center for Infertility affiliated to Shahid Sadoughi University of Medical Sciences and was approved by the ethics committee of Yazd Research and Clinical Center for infertility. 

**Figure 1:**
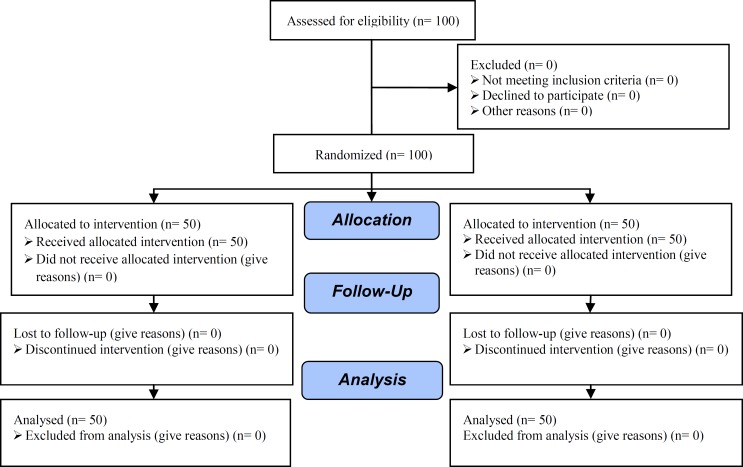
Consort flow diagram

A signed informed consent was obtained from all of the patients who participated in the study. A total of 100 patients indicated for ART were included in this prospective randomized clinical trial as consort flow chart (figure 1). The patients were randomly divided into two groups by using packets which included Computerized randomization. 

The inclusion criteria was as follows: female age between 18-38 years, history of infertility at least for 1 year, and FSH concentration in day 3 of menstrual cycle <12 mIU/ml. The patients with history of pelvic surgery, abnormal thyroid function or other endocrinopathies, and severe male factor (azoospermia) were excluded from the study. 

**Ovarian stimulation protocol**

All patients underwent pituitary desensitization by the administration of GnRH agonist on day 21 of the preceding menstrual cycle. In long-acting GnRH agonist group (group I), half-dose (1.87 mg) of Triptorelin (Diphereline® S.R. 3.75mg, IPSEN, Pharma, France) was administrated in a single intramuscular injection in mid-luteal phase (day 21) of the previous menstrual cycle. In short acting group (group II), Decapeptyl (Decapeptyl® 0.1 mg, Ferring Co., Germany) was started 0.1 mg per day subcutaneously from previous mid-luteal phase and continued until the day of HCG injection. 

Ovarian stimulation was done from day 2 of menstrual cycle with daily administration (100-150 IU) of human recombinant follicle-stimulating hormone (Gonal-f, Serono Co., Aubnne, Switzerland) and continued until the day of HCG injection. Ovarian response was monitored using serial ultrasound examination. When the leading follicle was larger than 18mm in diameter or at least two follicles were larger than 16mm, 10000 IU HCG (pregnyl, ® organon, oss, Netherlands) was injected intramuscularly. Oocyte was retrieved 36 hours after HCG injection using a 17-gauge aspiration needle under trans-vaginal ultrasound guidance. Oocyte was pre incubated in the medium at 37^o^C with 6% CO_2_ for 4-6 hours and inseminated by conventional IVF or intracytoplasmic sperm injection (ICSI). The fertilization was confirmed when two polar bodies and two pronuclear were observed 18-20 hours after insemination and 1-3 embryos were transferred 2-3 days after oocyte retrieval. 

The luteal phase support was initiated from the day of oocyte retrieval with 100 mg Progesterone in oil (Progesterone, Abureihan Co., Tehran, Iran) per day. Serum βhCG was measured after 14 days of embryo transfer. The clinical pregnancy was confirmed by observation of the fetal heart activity through trans-vaginal ultrasonography 4-5 weeks after oocyte retrieval. 

**Statistical analysis**

Statistical analysis was performed using the statistical package for the social science (SPSS software version 15.0 for windows, Chicago, IL). Both t-test and Chi-square test were used to detect significant differences (p<0.05) of the all variables between the two groups. 

## Results

A total of 100 patients were scheduled in this study (50 patients in each group). There were no differences in age, BMI, baseline FSH, etiology and duration of infertility between the two groups (Table I). 

There were also no differences in the number of Gonal-F used ampoules, estradiol level, and endometrial thickness on the day of HCG injection, and duration of stimulation between the two groups (Table II). The number of retrieved oocyte, total number of embryos, fertilization rate, implantation rate, and clinical pregnancy rate were similar in both groups (Table III). 

**Table I:** Basic characteristics of patients in long-acting and short-acting Decapeptyl groups

**Basic characteristics**	**Group I** **(Long-acting Decapeptyl)**	**Group II** **(Short-acting Decapeptyl)**	**p-value**
Age (Years)	28.35 ± 6.7	28.30 ± 4.7	0.927
BMI (Kg/m^2^)	25.00 ± 3.2	24.30 ± 3.6	0.557
Basal FSH (mIU/ml)	6.90 ± 2.6	6.10 ± 2.5	0.132

BMI: Body Mass Index. P-value<0.05 was significant. Student t test and Mann-Whitney test as appropriate

**Table II:** Etiology of infertility in long-acting and short-acting Decapeptyl groups

**Etiology**	**Group I** **(Long-acting Decapeptyl)**	**Group II** **(Short-acting Decapeptyl)**	**p-value**
Tubal factor	5%	7.5%	0.979
Ovulatory factor	35%	37.5%	1.000
Endometriosis	10%	5%	0.661
Unexplained	15%	10%	1.000
Male factor	35%	40%	0.910

P-value<0.05 was significant. Chi-square test

**Table III:** ART outcome in long-acting and short-acting Decapeptyl groups

**ART outcome**	**Group I** **(Long-acting Decapeptyl)**	**Group II** **(Short-acting Decapeptyl)**	**p-value**
No. of retrieved oocyte	8.35 ± 2.68	8.90 ± 3.00	0.545
Total No. of embryos	4.95 ± 1.66	5.25 ± 2.02	0.612
Fertilization rate (%) (Per cycle)	55.5	52.1	0.876
No. of transferred embryos	2.40 ± 2.5	2.45 ± 0.75	0.807
Implantation rate (%)	14	17	0.716
Clinical pregnancy rate (%) (Per cycle)	25	30	0.727

P-value<0.05 was significant. Mann-Whitney test and Chi-square test

## Discussion

In the present study, we compared two hypothalamic down-regulation/COH protocols; the half dose of depot form of Triptorelin and short-acting form of Decapeptyl. No difference was observed regarding number of retrieved oocyte and embryo and fertilization, implantation and pregnancy rates in both groups. In spite of our study, a recently-published analytical study has concluded that duration of stimulation and total number of FSH ampoules is significantly higher in depot form GnRH agonist group compared to short acting GnRH agonist group (10). 

Total doses of gonadotropin are so important that should be taken into careful consideration by both physicians and patients when choosing the type of protocol and it can change the cost of COH (10-13). Dal pratu *et al* compared half dose (1.87 mg) and full dose (3.75 mg) of depot Triptorelin. They found no significant difference in pregnancy and implantation rates between the two groups, but their results indicated that the number of administrated FSH ampoules were lower in half dose Triptorelin group (1).

In a similar study Yim *et al* concluded that half-dose of GnRH agonist was as effective as full-dose of GnRH-a. Owing to lower dose of HMG ampoules used, they recommended half-dose of GnRH in order to reduce the cost of treatment (2). Although our results showed that pregnancy rates were similar in both groups. Yael Gonen *et al* proved the superiority of short-acting GnRH agonist over long-acting agonist in achievement of pregnancy outcome (14). 

In some other studies, long-acting and short-acting GnRH agonist were compared and showed similar ART outcome in two types of GnRH agonist. Therefore, depot form was recommended in ART cycles for patients’ convenience (4, 5, 7, 8, 15, 16) .Depot form of GnRH agonist has a longer half-life and blocks GnRH receptors for up to 4 weeks after a single injection (8). Thus, there is potential risk of embryo exposure in early pregnancy. Lahat *et al* reported a high incidence of attention deficit hyperactivity disorder in long term follow-up of children exposed to GnRH agonist in early pregnancy (17). However, Tarlatzis reported that the rate of abortion and the health of children born after ART were not affected by the use of GnRH agonist protocol, altough some protocols were different success rate (18-20). Based on our findings, we recommend single dose depot form GnRH agonist for ART cycles, although its long-term effects on children require further investigation.

## Conclusion

No advantage was found concerning implantation and pregnancy rate and number of retrieved oocyte between half-dose long-acting and short-acting GnRH agonist groups in ART cycle.
